# Fabrication of a Capacitive 3D Spacer Fabric Pressure Sensor with a Dielectric Constant Change for High Sensitivity

**DOI:** 10.3390/s24113395

**Published:** 2024-05-24

**Authors:** Ji-Eun Lee, Sang-Un Kim, Joo-Yong Kim

**Affiliations:** 1 Department of Materials Science and Engineering, Soongsil University, Seoul 06978, Republic of Korea; leejjang8121@naver.com; 2 Department of Smart Wearable Engineering, Soongsil University, Seoul 06978, Republic of Korea; tkddnsl0723@naver.com

**Keywords:** wearable sensor, capacitive-type sensor, dip-coating, modulus, gauge factor, nonlinear regression, multilayer perception (MLP)

## Abstract

Smart wearable sensors are increasingly integrated into everyday life, interfacing with the human body to enable real-time monitoring of biological signals. This study focuses on creating high-sensitivity capacitive-type sensors by impregnating polyester-based 3D spacer fabric with a Carbon Nanotube (CNT) dispersion. The unique properties of conductive particles lead to nonlinear variations in the dielectric constant when pressure is applied, consequently affecting the gauge factor. The results reveal that while the fabric without CNT particles had a gauge factor of 1.967, the inclusion of 0.04 wt% CNT increased it significantly to 5.210. As sensor sensitivity requirements vary according to the application, identifying the necessary CNT wt% is crucial. Artificial intelligence, particularly the Multilayer Perception (MLP) model, enables nonlinear regression analysis for this purpose. The MLP model created and validated in this research showed a high correlation coefficient of 0.99564 between the model predictions and actual target values, indicating its effectiveness and reliability.

## 1. Introduction

Wearable sensors play an important role in various areas of our daily lives, including health care, convenience, and research. Through wearable sensors, data such as exercise amount [1,2,3], heart rate [4,5,6], respiration [7,8,9], and pulse [10,11,12] can be monitored in real time. Therefore, research and development related to wearable sensors are being increasingly emphasized.

First, since wearable sensors are worn directly on the skin, they must be made of a stretchable [13,14] and soft [15] material so that they can be worn for a long period of time without causing irritation or discomfort. Second, they should be relatively lightweight to allow unrestricted movement. Lastly, breathable materials are essential [16]. Therefore, various fabric-based sensors that meet the characteristics of wearable sensors are being studied. Many studies emphasize the necessity of textile sensors for biological signals and highlight the importance of flexible and elastic materials [17,18,19,20].

Sensors that measure subtle biological signals need to be easily deformable, even under low pressure. These sensors must have high sensitivity. Therefore, the more sensitive a sensor is to changes in electrical signals, the better it responds to minute pressure changes, significantly altering its electrical characteristics. Gauge factor (GF) is a critical factor in evaluating the sensitivity and performance of such sensors, and the equation for calculating GF is defined as follows [21]:

(1) $$GF=\frac{\Delta C/C_{0}}{\varepsilon }=\frac{\Delta C/C_{0}}{\Delta L/L_{0}}$$

where $C_{0}$ and $L_{0}$ are the initial capacitance and length, and ΔC and ΔL are the changes in capacitance and length after deformation. Sensors with a high gauge factor (GF) exhibit the ability to show significant changes in electrical properties even with minor pressure, enabling them to quickly detect slight changes. The more sensitive the sensor is, the more accurate and reliable the results are, and it can be used in a variety of fields.

There are two main types of sensors: resistive-type and capacitive-type. Resistive-type sensors have high hysteresis because their resistance changes with applied pressure. They also have a slow response time and high sensitivity due to their reaction to large changes. In contrast, capacitive-type sensors operate by measuring changes in the capacitance between electrodes, thereby detecting changes in electrical characteristics. As a result, they tend to function as less sensitive sensors compared to resistive-type sensors. However, this results in faster response times [22,23,24] and lower hysteresis [25,26,27]. Therefore, various studies are suggesting ways to enhance the sensitivity of capacitive-type sensors to make them more effective for use as sensors [28,29].

Capacitance can be calculated using the following equation [30]:

(2) $$C=\varepsilon_{r}\varepsilon_{0}\frac{A}{d}$$

where C is the capacitance, $\varepsilon_{r}$ is the relative dielectric constant, $\varepsilon_{0}$ is the dielectric constant of air, and A is the electrode plate area, and d is the distance between the electrode plates. According to Equation (2), capacitance changes when the distance (d) changes due to pressure applied to the sensor. This distance change relates to the modulus characteristics, which define the material’s stiffness. For a single material, once its modulus and dielectric constant are set, only the distance between electrodes varies, affecting capacitance. Thus, GF remains unchanged as capacitance varies proportionally with the degree of deformation. However, for composite materials, the dielectric constant changes with the distance between electrodes when compressed. If this distance decreases under pressure, the dielectric constant alters, creating an electrical signal. Research on changing dielectric properties using composite materials is actively being conducted. A study showed that increasing carbon nanotube (CNT) concentrations in spandex fabric raises its dielectric constant [31]. In addition, Wang et al. developed a dielectric material with a high dielectric constant by preparing a composite in which ionic liquids are uniformly dispersed in a PVDF material at nanoscale [32]. Various studies have been conducted to measure changes in the dielectric constant by applying ‘pressure’ as well as changes in composite materials. For example, Rathod et al. demonstrated that pressure affects the dielectric properties of GO-doped PVA nanocomposites, suggesting their use in pressure sensing applications [33]. This study emphasizes the sensitivity of composite materials to pressure-induced changes. In this way, it is found that the change in dielectric properties according to pressure could be applied by controlling the amount of conductive particles [34,35]. Research involving carbon nanotubes, a type of conductive particle, is being actively conducted [36,37].

**Figure 1:**
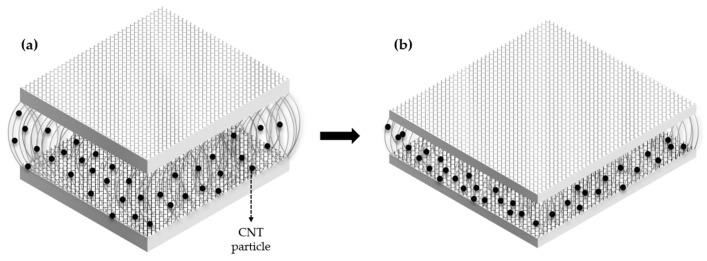
Structural diagram of CNT particle distribution before (**a**) and after (**b**) pressure application.

Overall, the objective of this study is to develop a capacitive-type sensor using 3D spacer fabric that is breathable and responsive to pressure, making it suitable for wearable applications. This fabric incorporates CNTs to enhance the change in dielectric constant, vital for sensor sensitivity. When pressure is applied, the concentration of CNT particles within the same space increases, causing the dielectric constant to change (Figure 1). Furthermore, the study utilizes a Multilayer Perceptron (MLP) model to efficiently predict the dielectric constant for various CNT weight percentages. This model accurately forecasts how the dielectric constant increases nonlinearly with pressure. Employing the MLP model significantly streamlines the research process by reducing the number of required experiments, enabling quicker and more cost-effective predictions of the sensor’s gauge factor.

## 2. Materials and Methods

### 2.1. Dip-Coating Process with Conductive Particle CNTs

**Figure 2:**
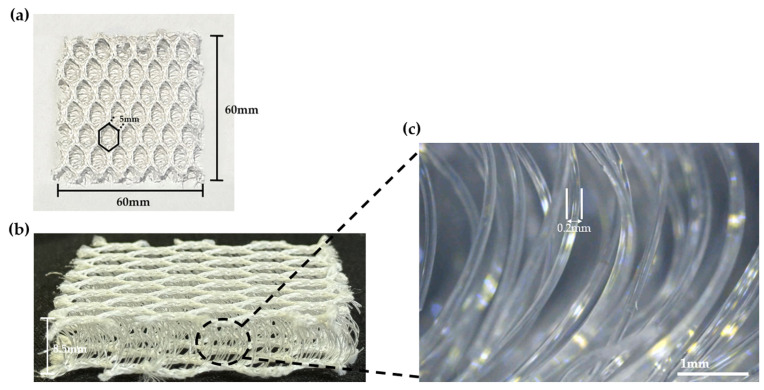
3D spacer fabric top layer (**a**), the filament used as the connecting yarn (**b**), and optical microscope image (**c**).

In this study, The base fabric is a Polyester (PE) 3D spacer fabric. The thickness of the fabric is 8.5 mm and the size is 60 mm × 60 mm. As shown in Figure 2, The structure of the spacer fabric consists of two mesh sides, and the PE monofilaments whose diameter is 2 mm sustain the structure between the mesh sides. The cross-section of the spacer fabric was also observed using an optical microscope as shown in Figure 2c.

**Figure 3:**
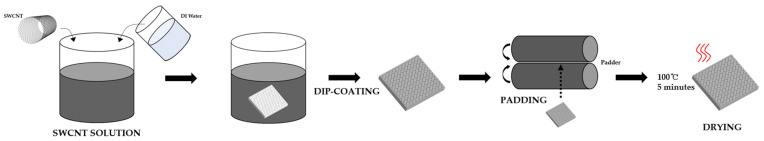
Schematic view of the CNTs particle dip-coating process and sample fabrication method.

**Figure 4:**
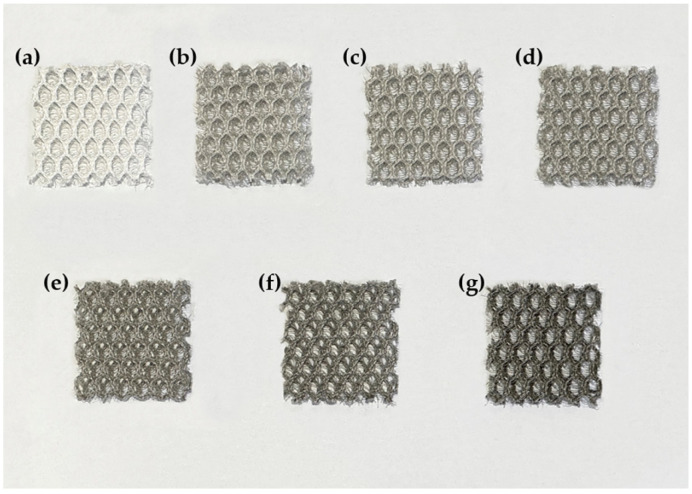
Color changes of (**a**) 0 wt%, (**b**) 0.005 wt%, (**c**) 0.01 wt%, (**d**) 0.02 wt%, (**e**) 0.04 wt%, (**f**) 0.06 wt%, (**g**) 0.08 wt% concentration fabrics.

Figure 3 shows the dip-coating process to attach the conductive particles to the PE monofilaments in the 3D spacer fabric. First, we used 0.1 wt% Single-Walled Carbon Nanotubes (SWCNTs, KORBON Co., Ltd., Gangwon-do, Republic of Korea) water dispersion and Di water to prepare 0.005, 0.01, 0.02, 0.04, 0.06, and 0.08 wt% dispersions. Second, The same size of 3D spacer fabrics dip-coated into the dispersion and padded 4 times of each fabric using a padder to prevent nonuniform coating of the SWCNTs on the surface. Finally, the coated fabrics dried for 5 min at 100 degrees using a drying oven. Figure 4 shows samples after the completion of the dip-coating process, demonstrating color changes as the concentration increases. Using a scanning electron microscope (the ZEISS Gemini SEM 300 from Oberkochen, Germany), it was confirmed that the SWCNTs were coated.

### 2.2. Measuring Instrument

#### 2.2.1. Mechanical and Electronic Properties

In this study, resistance is measured to determine the suitability of using the samples as capacitive-type sensors. A KEYSIGHT B2902A Source Measure Unit (SMU) for supplying current and a Universal Testing Machine (UTM) from Dacell Co. Ltd., Seoul, Korea for applying pressure were used. Copper tape is attached to the top and bottom of the fabric to form electrodes. Samples with attached electrodes are subjected to pressure at various concentrations using the UTM to measure resistance. Dou et al. showed that the electrical resistance decreased as the pressure increased by using a weight-knit spacer fabric in which CNT particles were dispersed [38]. Additionally, the modulus of the fabricated samples is also measured using the UTM, allowing for the analysis of both mechanical and dielectric properties. After fixing the sample, pressure is applied to compress it up to a 50% strain, and this process is measured ten times.

#### 2.2.2. Dielectric Properties

**Figure 5:**
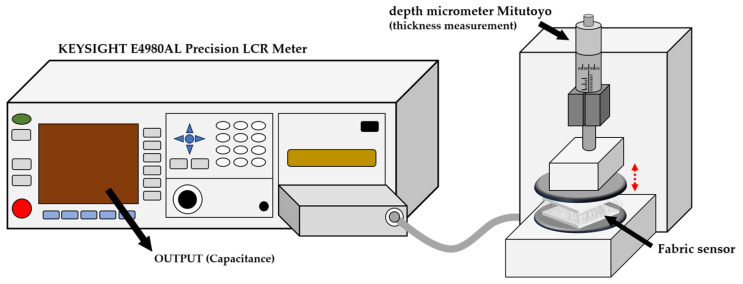
Measurement of capacitance using an LCR meter and micrometer.

The change in dielectric constant is linked to the sensitivity of the sensor. Therefore, capacitance measurements are used to determine the dielectric constant and derive its changes. In this study, a KEYSIGHT E4980AL Precision LCR Meter is connected with a micrometer to measure capacitance. Specifically, a depth micrometer (Mitutoyo Co., Tokyo, Japan) is utilized to apply strain to the sample based on pressure. Figure 5 shows the overall process of this measurement method. Using the LCR Meter, capacitance is measured at 300 Hz, and changes in capacitance are determined. Subsequently, calculations are made to find changes in the dielectric constant based on CNT content and pressure. Singh et al. defined the equation to calculate the dielectric constant [39]:

(3) $$\varepsilon =\frac{C_{p}}{C_{0}}$$

where $C_{p}$ is the capacitance of the manufactured sample, and $C_{0}$ is the capacitance measured when only air is present. Using Equation (3), the dielectric constant can be calculated.

#### 2.2.3. MLP Dielectric Constant Prediction

**Figure 6:**
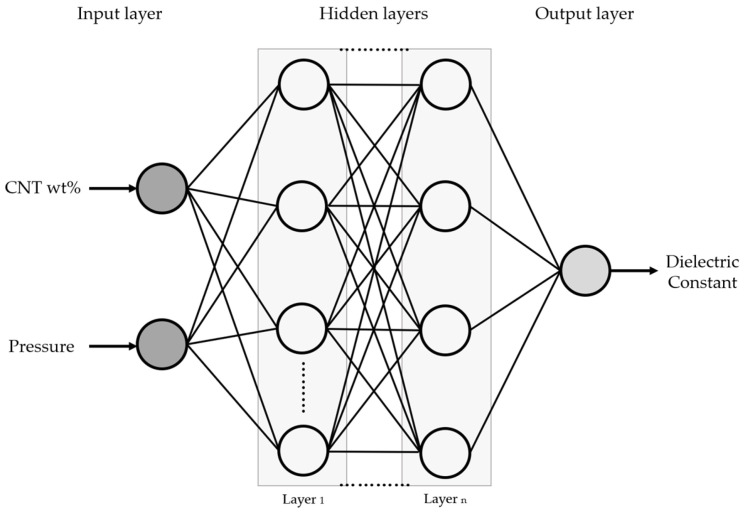
Multilayer Perceptron neural network.

The MLP (Multilayer Perceptron) neural network is utilized to conveniently deduce the dielectric constant values at the desired concentrations. The MLP model is structured as follows (Figure 6): First, in the input layer, the primary variables of the manufactured sensor, CNT wt% and pressure, are introduced. Second, the hidden layer processes data received from the input layer and performs various transformations internally before passing it on to the output layer. This layer enables the model to learn complex features and patterns from the data. Finally, the output layer predicts the dielectric characteristics of the sensor.

Using MATLAB programming software (R2023a), a MLP neural network model is created with the collected data, and this model is trained and validated. 

## 3. Results and Discussion

### 3.1. Fabrication and Characterization of CNT Particle-Based Spacer Fabric

**Figure 7:**
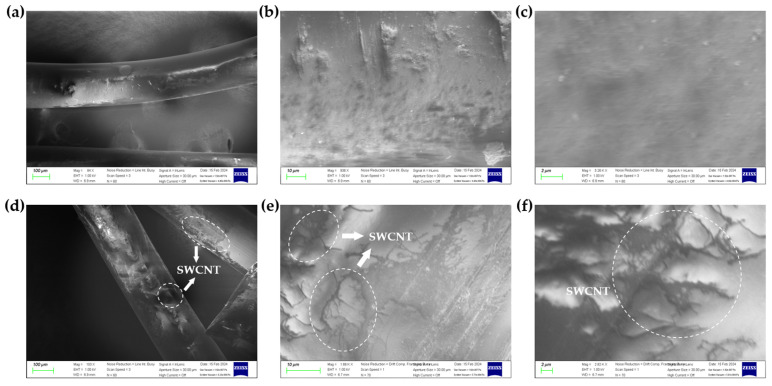
SEM images 0 wt% magnification 100 μm (**a**), 10 μm (**b**), 2 μm (**c**), and 0.08 wt% magnification 100 μm (**d**), 10 μm (**e**), 2 μm (**f**).

Figure 7 shows the fabric SEM (scanning electron microscope) morphology before and after dip-coating with SWCNT. Figure 7a–c show SEM images showing that the polyester-based fabric filament is smooth surfaces before coating. On the other hand, Figure 7d–f show that the filament surface is covered with SWCNT.

### 3.2. Electrical Resistance Based on the wt% and Pressure Variables of SWCNT Dispersion

Resistance measurement using an LCR meter is presented in Table 1. Resistance was measured by applying pressure, and the percentage values related to pressure presented in Table 1 are based on the strain of the 3D spacer fabric. An ‘overflow’ (OF) in electrical resistance indicates that the material possesses a very high intrinsic resistance. The measuring range of an LCR meter is from 2 Ω to 200 MΩ. For the 0.06 wt% sample, the resistance was not measured up to 30% pressure, but it showed that the resistance of 0.53 MΩ was measured when 40% pressure was applied. As the concentration and pressure on the sample increase, contacts between CNTs also increase, creating more conductive pathways and thus lowering the resistance. Notably, for the 0.08 wt% sample, resistance decreased significantly as pressure increased from 40% to 50%. This suggests the formation of a conductive network among CNTs, a phenomenon that percolation theory explains, where compression increases the fraction of CNTs in the same volume. Samples up to 0.04 wt%, which maintain high resistance across all pressure values, are suitable for capacitive-type sensors.

### 3.3. Calculating Dielectric Constant and Mechanical Property

The dielectric constant based on the capacitance of 3D spacer fabric samples from 0 wt% to 0.04 wt% is shown in Table 2. Samples dispersed with CNT particles tend to show an increase in capacitance and dielectric constant as their concentration and pressure levels increase.

**Figure 8:**
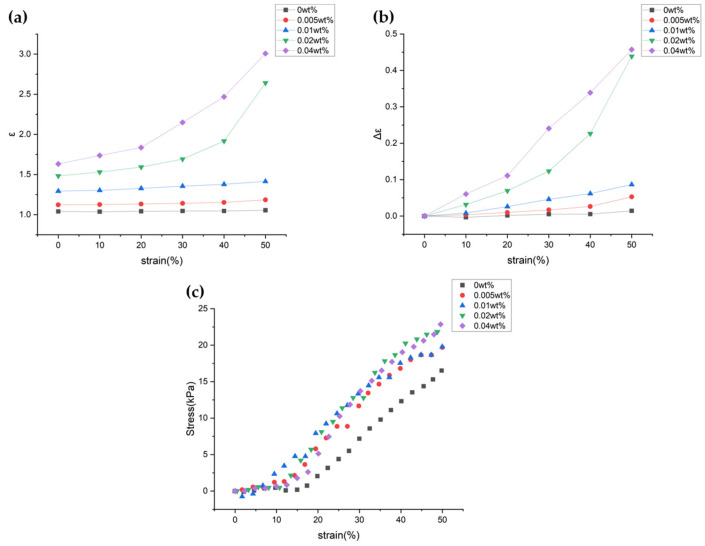
Graph of dielectric constant (**a**) and graph of changes in dielectric constant (**b**) based on pressure and CNT wt%; Modulus of the samples (**c**).

First, Figure 8a shows that increasing CNT wt% under constant pressure leads to a rise in dielectric constant (ε), attributed to the inherent electrical properties of CNTs. As the wt% of CNTs increases, the contact between CNT particles and interfacial polarization within the fabric increases, leading to a rise in the dielectric constant. Second, in fabrics dispersed with CNT particles, an increase in pressure tends to raise the dielectric constant. This effect occurs as the distance between CNT particles within the fabric decreases and their contact increases. However, at the initial pressure, the change in dielectric constant is minimal, which is related to the modulus. Figure 8c shows the stress according to pressure, where the slope represents the modulus. Since 3D spacer fabric is a porous material, the air layers escape first when pressure is applied, resulting in little change in modulus at initial pressures. After the air layers escape, as the pressure increases, the modulus also increases, and the modulus after 10% pressure is about 40–60 kPa. This indicates that the material is soft and sensitive to even small changes. Particularly for the 0.02 wt% and 0.04 wt% samples, there is a significant change in the dielectric constant at pressures above 30% (Figure 8b). As the concentration of CNT particles increases, the particles are more closely and effectively arranged, enhancing the dielectric properties of the dielectric. Additionally, due to the excellent tensile strength of CNTs, samples with dispersed CNT particles exhibit a slightly increased modulus compared to the 0 wt% CNT sample.

**Figure 9:**
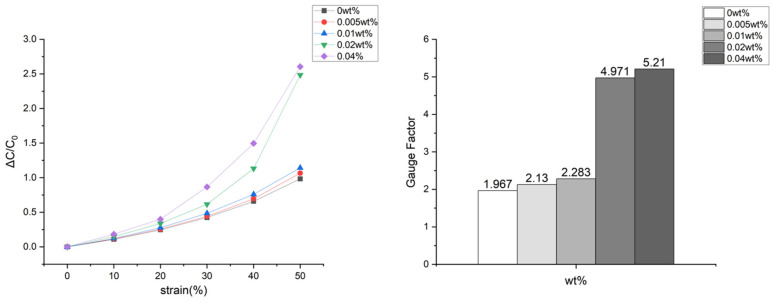
Graph of gauge factor Changes According to Each CNT Weight Percentage.

Gauge factor (GF) is a critical parameter determining the sensitivity of sensors. Figure 9 shows a steep increase in the slope within the 3D spacer fabric as the CNT wt% and pressure increase. The average GF for spacer fabric without CNT coating is 1.967. For spacer fabric coated with 0.005 wt%, it’s 2.130; for 0.01 wt%, it’s 2.283; for 0.02 wt%, it’s 4.971; and for 0.04 wt%, it’s 5.210. This increase in GF indicates greater sensitivity to pressure, enabling more accurate measurements. Especially for 0.02 wt% and 0.04 wt%, the electrical properties of CNT cause GF to increase significantly under higher pressures. Determining the optimal CNT wt% is crucial, depending on the application.

### 3.4. Evaluation of the MLP Dielectric Constant Prediction Model

**Figure 10:**
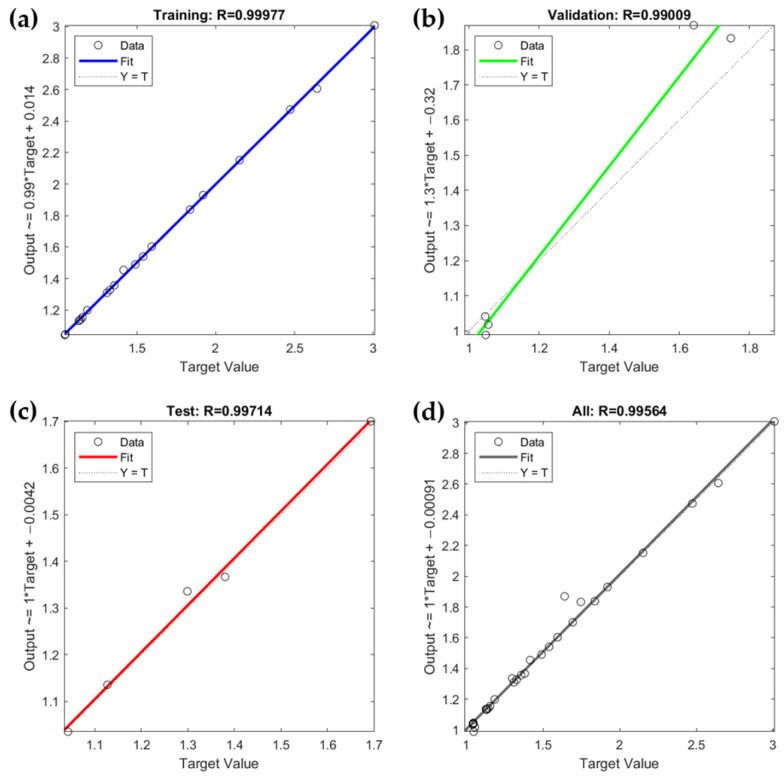
Correlation coefficient (R) graphs in Training (R = 0.99977) (**a**), Validation (R = 0.99009) (**b**), Test (R = 0.99714) (**c**), and All Stages (R = 0.99564) (**d**).

The implemented MLP model is evaluated through correlation coefficients in the ‘Training’, ‘Validation’, ‘Test’, and ‘All’ phases, as shown in Figure 10. High correlation coefficients indicate strong agreement between the network’s predictions and actual target values. The Training phase (Figure 10a) has a coefficient of 0.99977, showing excellent training data fit. The Validation phase coefficient is 0.9909 (Figure 10b), indicating good generalization. The Test phase (Figure 10c) shows a coefficient of 0.99714, reflecting high accuracy with test data. Finally, the ‘All’ phase (Figure 10d) shows a coefficient of 0.99564, demonstrating the model’s overall reliability. This MLP neural network simplifies modeling by reducing the need for complex experiments.

**Figure 11:**
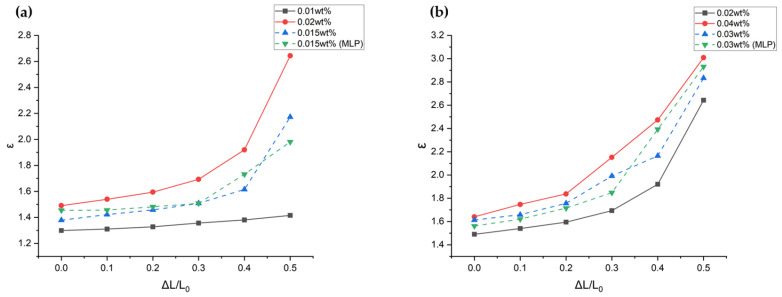
A comparison graph of the dielectric constants measured through MLP and experiments under conditions of 0.015 wt% (**a**) and 0.03 wt% (**b**).

The developed MLP model predicted dielectric constants for 0.015 wt% and 0.03 wt% data values, reducing the need for experimental procedures. However, in order to verify once more that the prediction is accurate, 0.015 wt% and 0.03 wt% SWCNT dip-coating processes were additionally performed, and the capacitance of the resulting samples was measured. Table 3 compares the experimentally measured dielectric constants ($\varepsilon_{E}$) with the MLP-predicted values ($\varepsilon_{M}$). Graphs were generated to intuitively verify if the experimental and theoretical data match. Figure 11a shows that the dielectric constant for 0.015 wt% fits well between 0.01 wt% and 0.02 wt% across all pressure conditions, indicating minimal error. Similarly, Figure 11b demonstrates that the dielectric constant for 0.03 wt% samples falls between 0.02 wt% and 0.04 wt%, also with low error.

Additionally, the regression performance of the MLP model was evaluated using the Mean Absolute Error (MAE) method. MAE calculates the average of the absolute values of the errors, thereby assigning equal weight to all errors. The equation for calculating MAE is as follows [40]:

(4) $$MAE=\frac{1}{m}\sum_{i=1}^{m}\left|X_{i}-Y_{i}\right|$$

The calculated errors for the 0.015 wt% and 0.03 wt% samples from Table 3 are, respectively, 0.07388 and 0.09998, indicating minimal error and high accuracy of the MLP model. Despite the nonlinear dielectric constant plot from the experiment, the MLP predictions matched the experimental data well. This suggests that the model predicts with a small error and performs accurate nonlinear regression analysis. Thus, this MLP model is effective for calculating the gauge factor in various sensor applications.

## 4. Conclusions

With the growing emphasis on the convenience of wearable sensors, research aimed at meeting their specific requirements has become increasingly significant. This study focuses on developing a highly sensitive capacitive-type sensor by dispersing Carbon Nanotube (CNT) particles in a polyester-based 3D spacer fabric. The fabricated sensor is adept at accurately measuring subtle physiological signals such as human movement, body temperature, and heart rate.

Samples with a maximum CNT concentration of 0.04 wt% were selected because those with measurable resistance are not suitable for Capacitive-type sensors. To use polyester-based fabric as a sensor, it needs to exhibit a high dielectric constant change to create a highly sensitive sensor. When various concentrations of CNT dispersion were dip-coated, samples with 0.02 wt% and 0.04 wt% showed steep changes in dielectric constant with pressure. This is due to the increased contact between conductive particles, and the change in dielectric constant correlates with the Gauge Factor, an indicator of sensor sensitivity. The measurements showed very sensitive values, with GF = 4.971 at 0.02 wt% and GF = 5.210 at 0.04 wt%. Additionally, all samples had a modulus between 40–60 kPa, proving that the sensor material is soft and responsive to pressure, thus highly sensitive. These characteristics are very important for the application of wearable sensors and ensuring high performance in real-world environments. Lastly, the dielectric constant was efficiently predicted without the hassle of experimental processes using a Multilayer Perceptron (MLP) for artificial intelligence nonlinear regression. Since many biological signals require their respective suitable gauge factors, applying this model can aid in creating textile pressure sensors more suitable for wearable devices.

To advance this research, the sensitivity and flexibility of the sensor can be further enhanced by using a variety of composite materials that meet the sensor’s requirements. Additionally, the practicality of the sensors will be validated through performance evaluations in real-world settings. The developed sensors will enhance accuracy through more complex MLP processes and expand the application range of wearable sensors.

## Figures and Tables

**Table 1:** Resistance based on CNT weight percentage (wt%) and pressure. (The dash (-) represents ‘Overflow(OF)’).

	Resistance (MΩ)						
Strain	0 wt%	0.005 wt%	0.01 wt%	0.02 wt%	0.04 wt%	0.06 wt%	0.08 wt%
0%	-	-	-	-	-	-	7.72
10%	-	-	-	-	-	-	6.26
20%	-	-	-	-	-	-	1.96
30%	-	-	-	-	-	-	1.12
40%	-	-	-	-	-	0.53	0.24
50%	-	-	-	-	-	0.31	0.06

**Table 2:** Measurement of capacitance (unit: pF) and dielectric constant.

		Capacitance (pF), Dielectric Constant					
Strain	$C_{p}$ & ε	Air	0 wt%	0.005 wt%	0.01 wt%	0.02 wt%	0.04 wt%
0%	$C_{p}$	1.166	1.213	1.308	1.507	1.729	1.903
	ε		1.040	1.122	1.292	1.4983	1.632
10%	$C_{p}$	1.297	1.345	1.460	1.690	1.986	2.253
	ε		1.037	1.126	1.303	1.531	1.737
20%	$C_{p}$	1.451	1.512	1.644	1.925	2.312	2.664
	ε		1.042	1.133	1.327	1.593	1.836
30%	$C_{p}$	1.652	1.727	1.885	2.238	2.794	3.551
	ε		1.045	1.141	1.355	1.691	2.150
40%	$C_{p}$	1.924	2.12	2.238	2.650	3.687	4.749
	ε		1.046	1.152	1.377	1.916	2.468
50%	$C_{p}$	2.281	2.406	2.701	3.227	6.026	6.860
	ε		1.055	1.184	1.415	2.642	3.007

**Table 3:** Dielectric constant ($\varepsilon_{M}$ is the dielectric constant predicted through MLP, $\varepsilon_{E}$ and is the dielectric constant measured through experiments).

	Dielectric Constant $\varepsilon_{M}$$\;(\varepsilon_{E})$	
Strain	0.015 wt%	0.03 wt%
0	1.454 (1.378)	1.561 (1.613)
0.1	1.456 (1.422)	1.621 (1.658)
0.2	1.481 (1.457)	1.715 (1.756)
0.3	1.508 (1.509)	1.848 (1.992)
0.4	1.732 (1.615)	2.393 (2.165)
0.5	1.981 (2.172)	2.931 (2.833)

## Data Availability

Data are contained within the article.
